# Cytoplasmic LXR expression is an independent marker of poor prognosis for patients with early stage primary breast cancer

**DOI:** 10.1007/s00432-021-03670-y

**Published:** 2021-06-03

**Authors:** Wanting Shao, Christina Kuhn, Doris Mayr, Nina Ditsch, Magdalena Kailuwait, Verena Wolf, Nadia Harbeck, Sven Mahner, Udo Jeschke, Vincent Cavaillès, Sophie Sixou

**Affiliations:** 1 Department of Obstetrics and Gynecology, Breast Center, LMU University Hospital, Munich, Germany; 2 Department of Breast Center, School of Medicine, The First Affiliated Hospital, Zhejiang University, Hangzhou, 310003 China; 3 Department of Gynecology and Obstetrics, University Hospital Augsburg, Stenglinstr. 2, 86156 Augsburg, Germany; 4 Department of Pathology, LMU Munich, Munich, Germany; 5 IRCM—Institut de Recherche en Cancérologie de Montpellier, INSERM U1194, Université Montpellier, Parc Euromédecine, 208 rue des Apothicaires, F-34298 Montpellier, France; 6 Faculté Des Sciences Pharmaceutiques, Université Toulouse III - Paul Sabatier, 31062 Toulouse, France; 7 Cholesterol Metabolism and Therapeutic Innovations, Cancer Research Center of Toulouse (CRCT), UMR 1037, INSERM, UPS, Université de Toulouse, CNRS, 31037 Toulouse, France

**Keywords:** Breast cancer, Early stage, Prognostic marker, LXR, Subcellular location

## Abstract

**Purpose:**

The aim of this study was to investigate the expression of liver X receptors α/β (LXR) in primary breast cancer (BC) tissues and to analyze its correlations with clinicopathological parameters including patient survival.

**Methods:**

In a well-characterized cohort of 305 primary BC, subcellular distribution of LXR was evaluated by immunohistochemistry. Correlations with clinicopathological characteristics as well as with patient outcome were analyzed.

**Results:**

LXR was frequently localized in both nuclei and cytoplasms of BC cells, with stronger staining in nuclei. Total and nuclear LXR expression was positively correlated with ER and PR status. Overall survival analysis demonstrated that cytoplasmic LXR was significantly correlated with poor survival and appeared as an independent marker of poor prognosis, in stage I but not in stage II–III tumors

**Conclusion:**

Altogether, these data suggest that cytoplasmic LXR could be defined as a prognostic marker in early stage primary BC.

**Supplementary Information:**

The online version contains supplementary material available at 10.1007/s00432-021-03670-y.

## Introduction

Breast cancer (BC) has surpassed lung cancer as the most frequent diagnosed cancer worldwide in 2020 and is still the leading cause of cancer death among women (Sung et al. 2021). Therapeutic strategies for BC are defined according to the immunohistochemical detection of tumor biomarkers which include estrogen receptor (ER), progesterone receptor (PR), epidermal growth factor receptor 2 (HER2), and Ki67 (Harbeck et al. 2019). Systemic therapy approaches, including endocrine therapy, anti-HER2 therapy, and chemotherapy, have achieved success in improving clinical outcomes of early BC patients (Harbeck and Gnant 2017; Pondé et al. 2019).

However, many early stage patients suffer long-term relapse or metastasis after routine treatments. Therefore, identification of novel biomarkers is necessary for advances in individualized and/or combined BC therapies. Among the early stage primary BC which is expected to have a good prognosis according to the above-mentioned markers, a special need exists for new markers to identify the subgroup of patients that will eventually relapse from their disease.

Beside ER and PR, other nuclear receptors (NR) play a role in BC, as we and other have reported earlier (Bock et al. 2014; Ditsch et al. 2012; Doan et al. 2017; Heublein et al. 2017; Jalaguier et al. 2017; Jeschke et al. 2019; Shao et al. 2020a, 2020b; Zhang et al. 2017). Liver X receptors (LXRs) belong to the NR superfamily (Wang and Tontonoz 2018) and have two isotypes, LXRα and LXRβ (encoded by the *NR1H3* and *NR1H2* gene, respectively). LXRα is highly expressed in adipose tissue, liver, adrenal glands, lungs, and gastrointestinal tract, while LXRβ is widely expressed (Repa and Mangelsdorf 2000). Upon activation by ligands, LXRs heterodimerize with retinoid X receptors (RXRs) and bind to target gene promoters, resulting in the regulation of various cellular parameters such as cholesterol synthesis and transport, glucose homeostasis, inflammatory, and immune responses (Bilotta et al. 2020; Lin and Gustafsson 2015).

LXR are also important actors in cancer biology. Indeed, previous studies revealed that natural LXR ligands (namely 25- and 27-hydroxycholesterol) play an important role in lung and breast cancer by promoting invasion, migration, and metastasis through an LXR-dependent pathway (Chen et al. 2017; Nazih and Bard 2020; Nelson et al. 2013). Nonetheless, studies based on LXR activation using the synthetic agonists T0901317 and/or GW3965 reported antineoplastic effects in various cancer types (Derangère et al. 2014; Lou et al. 2019; Pommier et al. 2010; Scoles et al. 2010; Vedin et al. 2009; Wang et al. 2019; Zhong et al. 2020). In various LXR-positive human BC cell lines, LXR agonists inhibited cell proliferation and increased p53 protein level (Vedin et al. 2009).

Moreover, LXR expression was reduced in liver and prostate cancers as compared to the adjacent normal tissues, indicating that LXR expression decreases during carcinogenesis (Chen et al. 2020; Long et al. 2018). However, little is known about LXR expression and its prognostic value in breast cancer.

In the present study, we analyzed LXRα/β expression by immunohistochemistry (IHC) in a well-characterized cohort of 305 primary BC patients. We quantified LXR levels both in the nuclear and cytoplasmic compartments, and analyzed correlations with clinicopathological parameters and patient survival.

## Materials and methods

### Patient cohort

All samples (*n* = 305, two cases from one patient with bilateral BC) in this retrospective analysis were collected at the Department of Obstetrics and Gynecology of the Ludwig-Maximilians-University Munich, Germany between 2000 and 2002. This study was approved by the Ethical Committee of the Medical Faculty, Ludwig-Maximilian-University, Munich, Germany (approval number 048-08; 18th of March 2008) and informed consent was obtained from all patients. Patient data obtained from the Munich Cancer Registry were pseudonymized and samples were encoded during experiments and statistical analysis. All clinical diagnostic and therapeutic procedures were completed before this study. All tumors were evaluated according to UICC TNM classification, including tumor size (pT), lymph-node involvement (pN), and distant metastasis (cM). Tumor grade was confirmed by an experienced pathologist (Dr. D. Mayr) of the LMU Department of Pathology, according to a modification of Elston and Ellis grading proposed by Bloom and Richardson. ER, PR, HER2, and Ki67 were determined by an experienced pathologist (LMU Department of Pathology) at first diagnosis using immunohistochemistry. For ER and PR staining, tissues showing nuclear staining in more than 10% of tumor cells were considered as hormone receptor-positive. HER2 expression was analyzed with an automated staining system (Ventana; Roche, Mannheim, Germany), according to the manufacturer’s instructions. Cases were regarded as negative for 0 or 1 + score, and positive for 3 + scores. All cases with 2 + scores needed a further evaluation, i.e., fluorescence in situ hybridization (FISH) testing. Ki67 cut-off used to differentiate luminal A from luminal B tumors (all HER2-negative) was 14% as commonly used at the time of diagnosis.

### Immunohistochemistry (IHC)

Immunohistochemical staining for LXR was performed as previously described (Jeschke et al. 2019; Müller et al. 2019; Shao et al. 2020a, 2020b; Sixou et al. 2018). Briefly, whole tissue sections were cut and prepared from paraffin-embedded BC samples using standard protocols. After deparaffinizing in xylol for 20 min, endogenous peroxidase reaction was blocked with 3% hydrogen peroxide in methanol. Next, slides were rehydrated with a series of descending alcohol dilution and then boiled in a pressure cooker for 5 min, and immersed in sodium citrate buffer. Phosphate-buffered saline (PBS) was used for all washes and sections were incubated in blocking solution (ZytoChem Plus HRP Polymer System Kit, ZYTOMED Systems GmbH, Berlin, Germany) before incubation with primary antibody against LXR (LS-B262, Lifespan Biosciences, WA, USA) with a 1:200 dilution for 16 h at 4 °C. The antibody used was raised against A synthetic peptide made to an internal portion of the human LXR protein sequence (between residues 50–150), resulting in specificity against both LXRα and LXRβ isoforms. After incubation with a biotinylated secondary anti-rabbit IgG antibody, and with the associated avidin–biotin–peroxidase complex (both Vectastain Elite ABC Kit; Vector Laboratories, Burlingame, CA, USA), visualization was performed with substrate and chromogen 3, 3-diamino-benzidine (DAB; Dako, Glostrup, Denmark). Negative and positive controls were used to assess the specificity of the immunoreactions. Negative controls (colored in blue) were performed in BC tissue by replacement of the primary antibodies by species-specific (rabbit/mouse) isotype control antibodies (Dako, Glostrup, Denmark). Appropriate positive controls (placenta samples) were included in each experiment. Sections were counterstained with acidic hematoxylin, dehydrated and immediately mounted with Eukitt (Merck, Darmstadt, Germany) before manual analysis with a Diaplan light microscope (Leitz, Wetzlar, Germany) with 25× magnification. Pictures were obtained with a digital CCD camera system (JVC, Tokyo, Japan).

The staining of LXR was assessed according to a semiquantitative immunoreactive score (IRS), determined by multiplication of the positive cell proportion score (0 = 0%, 1 = 1–10%, 2 = 11–50%, 3 = 51–80%, and 4 = 81–100% stained cells) and the staining intensity score (0 = negative, 1 = weak, 2 = moderate, and 3 = strong). As previously described for RIP140, PPARγ, and THRβ1 (Shao et al. 2020a, 2020b), LXR cytoplasmic and nuclear staining results were evaluated in parallel, with a separate determination of cytoplasmic IRS and nuclear IRS. Total IRS was calculated by the sum of cytoplasmic and nuclear IRS. For all other markers, staining and IRS were determined in the whole cells, without differentiation of nuclear and cytoplasmic staining. A total of hundred cells (three spots with around 30 cells each) were analyzed for each sample and the IRS corresponded to the mean of the IRS determined on the three spots by two independent blinded observers. Discordant cases were re-evaluated by both observers together. After re-evaluation, both observers agreed on the result.

### Statistical and survival analysis

Receiver-operating characteristic (ROC) curve analyses were performed to calculate the optimal cut-off values between low and high LXR expression, based on the maximum differences of sensitivity and specificity. The threshold determined regarding OS was an IRS > 2.5 for nuclear LXR, > 5 for cytoplasmic LXR and > 8.5 for total LXR, which were used to determine the percentages of tumors expressing low or high LXR levels described in Table 2. Correlation analyses presented in Tables 3 and 4 were performed by calculating the Spearman’s-Rho correlation coefficient (*p* values of Spearman’s-Rho test presented).

Survival times were compared by Kaplan–Meier graphics and OS differences were tested for significance using the chi-square statistics of the log-rank test. Data were assumed to be statistically significant in the case of *p* value < 0.05. Kaplan–Meier curves and estimates were then provided for each group and each marker. The *p* value and the number of patients analyzed in each group are given for each chart.

Statistical analyses above were performed using SPSS 25 (IBMSPSS Statistics, IBM Corp., Armonk, NY, USA). For all analyses, *p* values below 0.05 (*), 0.01 (**), or 0.001 (***) were considered statistically significant.

## Results

### Expression of LXR in primary BC tissues

LXR expression was assessed by IHC in a cohort of 305 primary BC samples (with clinical characteristics summarized in Table 1). We used an antibody directed to both LXRα and LXRβ isoforms, to focus on the importance of the subcellular expression of both isoforms. Among these samples, one bilateral primary BC was regarded as two individual cases. During follow-up, 40 patients have experienced a local recurrence, 58 have developed distant metastases, and 88 have died. Median age at initial diagnosis was 57.8 ± 0.7 years (range 26.7–94.6 years) and median follow-up time was 125 ± 38.6 months (range 1–153 months).

**Table 1:** Clinical and pathological characteristics of all patients

Clinical and pathological characteristics ^a^	*n* = 305^b^	%
**Age,** median (years)	57.88	
**Follow-up**, average (months)	110.22	
Median	125	
**Histology**^c^		
Invasive lobular	40	13.11
Invasive medullar	10	3.28
Invasive mucinous	4	1.31
No special type (NST)	160	52.46
DCIS with NST	79	25.90
Unknown	12	3.93
**Focality**		
Unifocal	167	54.75
Multifocal and/or multicentric	138	45.25
**ER status**		
Positive	246	80.66
Negative	59	19.34
**PR status**		
Positive	179	58.69
Negative	126	41.31
**HER2 status**		
Positive	35	11.48
Negative	268	87.87
Unknown	2	0.66
**Molecular subtype (IHC)**		
Luminal A (Ki67 ≤ 14%)	169	55.41
Luminal B (Ki67 > 14%)	61	20.00
HER2 positive	26	8.52
HER2 non-luminal	8	2.62
Triple negative	39	12.79
Unknown	2	0.66
**Grade**		
I	15	4.92
II	102	33.44
III	45	14.75
Unknown	143	46.89
**Staging**		
Stage I	135	44.26
Stage II	138	45.24
Stage III	16	5.25
Unknown	16	5.25
**Tumor size**		
pT1	192	65.31
pT2	86	29.25
pT3	4	1.36
pT4	12	4.08
Unknown	11	3.61
**Lymph-node metastasis**		
Yes	125	40.98
No	164	53.77
Unknown	16	5.25
**Local recurrence**		
Yes	40	13.11
No	254	83.28
Unknown	11	3.61
**Distant metastases**		
Yes	58	19.02
No	236	77.38
Unknown	11	3.61

^a^All information given refers to the primary tumor

^b^One of 304 patients are bilateral primary BC, so we deal with the tumor as individual one (*n* = 305)

^c^NST include the formerly called “Invasive ductal” and “other” types; DCIS is for Ductal carcinoma in situ

**Fig. 1:**
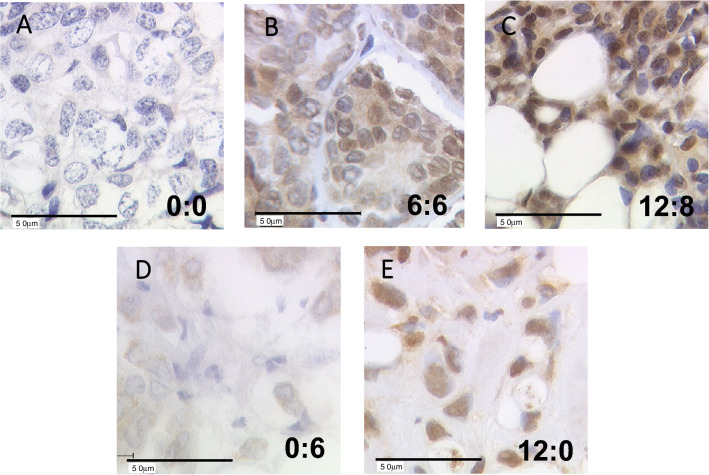
Immunohistochemical staining of LXR in BC samples. LXR staining is illustrated for five patients with absent, intermediate, or high LXR expression. Examples of tumors with opposite nucleo-cytoplasmic expression of LXR are given in panels **D** and **E**. Nucleo:cytoplasmic IRS (immunoreactive score) ratios are indicated in each photomicrograph (×25 magnification) and scale bar equals 50 μm

LXR immunoreactivity was present in both nucleus and cytoplasm of cancer cells. Examples of LXR staining from 5 patients are displayed in Fig. 1, with nucleo:cytoplasmic immunoreactive score (IRS) ratio indicated in each panel. Extreme (0:0 or 12:8) and intermediate (6:6) IRS were shown in panels A–C all showing equivalent distribution of LXR staining in the nuclear and cytoplasmic compartments. Besides, tumors with low nuclear and high cytoplasmic LXR expression (0:6) or with high nuclear and low cytoplasmic IRS (12:0) were also observed and are exemplified in panels D and E, respectively.

Distribution and correlation of LXR expression were analyzed in the whole cohort (Table 2). The mean IRS values of nuclear and cytoplasmic LXR expression were 5.11 and 3.05, respectively, demonstrating that LXR expression was stronger in the nucleus than in the cytoplasm. Both nuclear and cytoplasmic LXR expression was detected in a majority of BC samples, since only 15.41% and 28.52% of the tumors expressed no detectable nuclear and cytoplasmic LXR, respectively (Table 2).

**Table 2:** Distribution and correlation of LXR expression

	Total	Nuclear	Cytoplasmic
Mean IRS ± SE	8.16 ± 0.27	5.11* ± 0.20	3.05 ± 0.15
IRS range	0–20	0–12	0–12
IRS cut-off	8.5	2.5	5
Negative expression	32 (10.49%)	47 (15.41%)	87 (28.52%)
Low expression	161 (52.79%)	80 (26.23%)	237 (77.70%)
High expression	144 (47.21%)	225 (73.77%)	68 (22.30%)
Correlation coefficient			
Nuclear LXR	0.819***	1.000	0.144*
Cytoplasmic LXR	0.653***	0.144*	1.000

IRS cut-offs for low and high expression (defined using ROC-curve analysis) were defined by performing an ROC-curve analysis for OS

Correlations were statistically significant for *p* < 0.05 (*) or *p* < 0.001 (***), using Spearman-Rho test using mean bilateral analysis

Based on IRS cut-offs defined by performing an ROC-curve analysis for OS, all patients were divided into low and high expression subgroups. Based on these nuclear and cytoplasmic LXR cut-off values, an inverse distribution of BC was noticed, with a majority of samples exhibiting a high nuclear IRS (73.77% of the tumors) and a low cytoplasmic one (77.70%). Total LXR was positively correlated with nuclear and cytoplasmic (*p* = 4.23 × 10^−75^ and 1.84 × 10^−38^, respectively), and a positive correlation (*p* = 0.012) was also detected between nuclear and cytoplasmic LXR expression.

### Correlation between LXR expression and clinical parameters

Using Spearman’s-Rho test, we analyzed the correlation between LXR expression and known clinicopathological features, including age, tumor size (pT), lymph-node status (pN), metastasis (cM), histology, stage, tumor grade, ER, PR, and HER2 status. As shown in Table 3, total and nuclear LXR expression were positively correlated with ER and PR status, whereas they were not correlated with other aggressive markers (pT, pN, cM, histology, stage, grade, and HER2 status). It should be noted that at the time of diagnosis, grading was not available for 143 noninvasive lobular carcinoma (classified in the NST group in Table 1). No significant associations were observed between cytoplasmic LXR and all parameters mentioned in Table 3.

**Table 3:** Correlation between LXR expression and clinicopathological markers

	Total	Nuclear	Cytoplasmic
Age	− 0.077	− 0.050	− 0.057
pT	− 0.049	− 0.027	− 0.032
pN	− 0.089	− 0.088	− 0.025
cM	− 0.025	− 0.014	− 0.040
Histology	0.015	0.043	− 0.042
Stage	− 0.055	− 0.006	− 0.059
Grade	− 0.146	− 0.154	− 0.036
ER	0.137*	0.172***	0.006
PR	0.116*	0.114*	0.037
HER2	0.016	− 0.040	0.083

Correlations are statistically significant for *p* < 0.05 (*) or *p* < 0.01 (**), using Spearman-Rho test

### Correlation between cytoplasmic LXR expression and patient OS

**Fig. 2:**
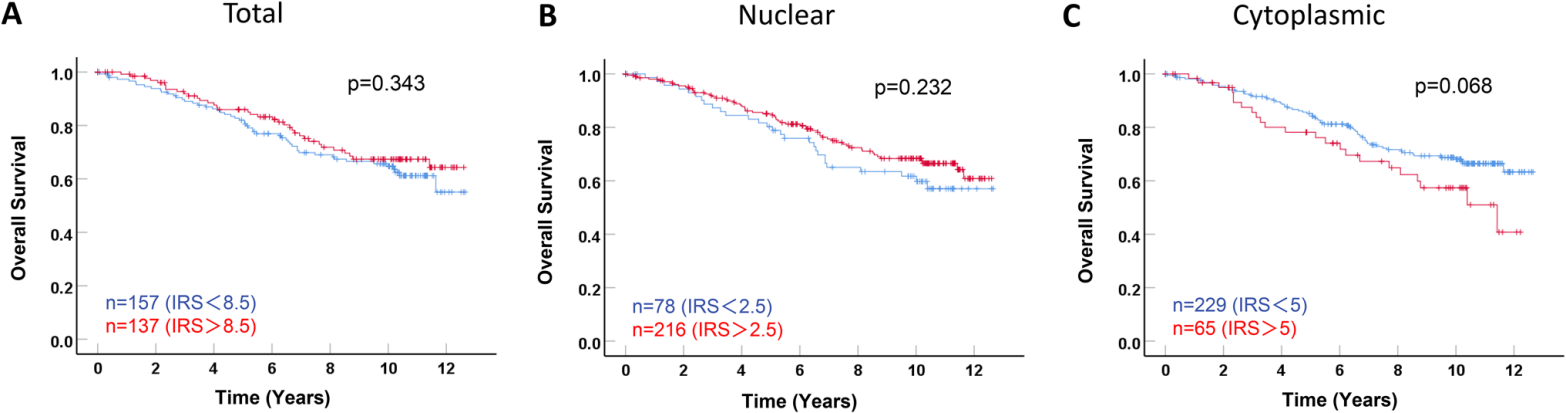
Kaplan–Meier analyses of patient overall survival (OS) in the whole cohort according to nuclear, cytoplasmic, and total LXR expression. OS Kaplan–Meier curves are presented according to total (**A**), nuclear (**B**), and cytoplasmic (**C**) LXR expression. The optimal IRS cut-off values for nuclear, cytoplasmic, and total LXR expression were determined as 2.5, 5, and 8.5, respectively. The number of cases for each group is indicated in each penal

As described in Table 2, we optimized the IRS cut-off values of LXR by performing ROC-curve analysis and divided the patient cohort into low and high expressing subgroups for all survival analyses. Comparisons of patient OS according to LXR expression levels were subsequently calculated by Kaplan–Meier analyses. Considering the whole cohort, no statistically significant correlations of nuclear, cytoplasmic, or total LXR expression were found with patient outcome (Fig. 2). Nonetheless, the trend existed with a possible favorable survival for the patients with low cytoplasmic LXR expression (*p* = 0.068).

**Fig. 3:**
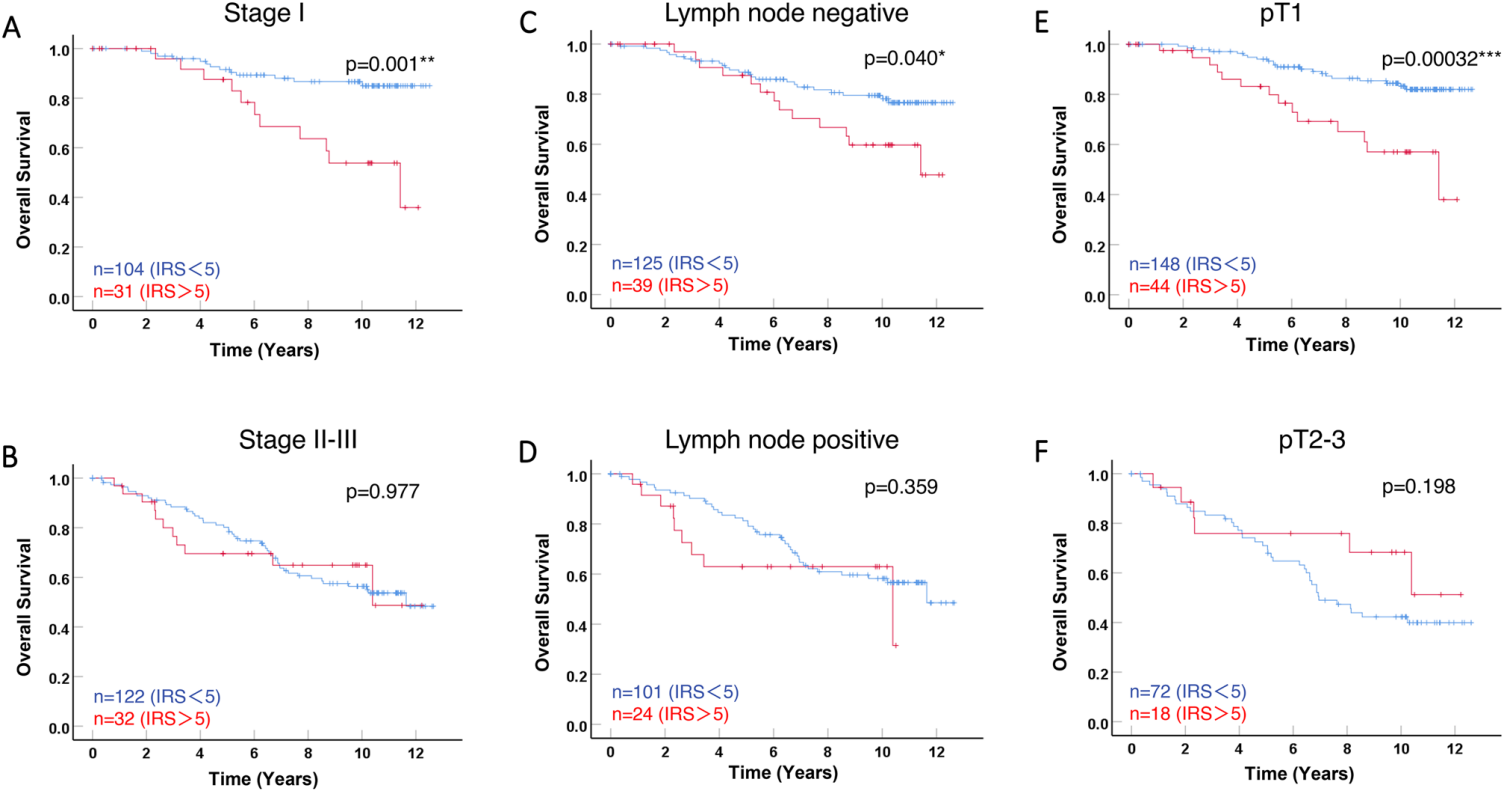
Kaplan–Meier analyses of patient overall survival for cytoplasmic LXR expression in subgroups according to tumor size, lymph-node status, and staging. Overall survival (OS) curves are presented according to cytoplasmic LXR, for Stage I (**A**) and stage II–III (**B**) subgroups of patients, for either lymph-node negative (**C**) or positive (**D**) subgroups, and for the pT1 (**E**) and pT2-3 (**F**). The optimal IRS cut-off value of 5 was used and the number of cases for each group is indicated in each panel. Statistical significance is shown as *p* value from log-rank test (*p < 0.05; ***p < 0.001)

We then then stratified patients in two subgroups, according to staging (stage I vs stage II–III). As shown in Fig. 3B, no significant difference was observed in the subgroups of patients with stage II–III (*p* = 0.977), whereas in stage I subgroup (Fig. 3A), patients with tumors showing a low level of cytoplasmic LXR expression had a significantly better outcome compared to those with a high level of cytoplasmic LXR (*p* = 0.001).

To better decipher this correlation with survival, we also stratify patients according to lymph-node status (negative vs positive) and tumor size (pT1 vs pT2-3). The correlation of cytoplasmic LXR with patient OS was significant in lymph-node negative tumors (Fig. 3C *p* = 0.040) but not in the subgroup of patients with tumors presenting lymph-node invasion (*p* = 0.359). The correlation was even more significant in the subgroup of patient with pT1 tumors (*p* = 0.00032, Fig. 3E) and lost in the pT2-3 subgroup (*p* = 0.198, Fig. 3F).

As a control, we also analyzed OS according to the expression of RXR, the heterodimerization partner of LXR. No significant correlations with OS were found in the whole cohort or in the subgroups according to staging, lymph-node status, or tumor size (Supplementary Fig. 1). Altogether, these data demonstrated that cytoplasmic LXR expression is associated with poor prognosis in patients with early stage BC (i.e., with small and noninvasive tumors).

### Cytoplasmic LXR expression as an independent prognostic parameter

Multivariate analyses were performed for the whole cohort and for the two subgroups according to staging (stage I vs stage II-III), using a Cox regression model with cytoplasmic LXR expression and other relevant clinicopathological characteristics, namely age at diagnosis, metastasis status, ER, and HER2 (Table 4). All parameters were demonstrated to be independent prognostic markers of OS for the whole cohort.

**Table 4:** Multivariate analysis of significant clinicopathological variables and of cytoplasmic LXR regarding OS in the whole cohort and in various subgroups

	Whole cohort (*n* = 279)			Stage I (*n* = 121)			Stage II-III (*n* = 145)		
	HR	95% CI	*p*	HR	95% CI	*p*	HR	95% CI	*p*
Age	1.055	1.037–1.074	2.95 × 10^-9***^	1.067	1.026–1.109	0.001**	1.049	1.028–1.071	5 × 10^6***^
cM	5.692	3.666–8.836	9.27 × 10^-15***^	18.632	6.197–56.014	1.91 × 10^-7***^	4.801	2.789–8.264	1.50 × 10^-8***^
ER	0.492	0.307–0.789	0.003**	0.248	0.093–0.659	0.005**	0.513	0.290–0.909	0.022*
HER2	1.824	1.033–3.220	0.038*	0.758	0.203–2.835	0.681	4.257	2.122–8.540	4.5 × 10^-5***^
Cytoplasmic LXR	1.871	1.156–3.030	0.011*	7.172	2.764–18.611	5.1 × 10^-5***^	0.918	0.462–1.826	0.808

*HR* hazard ratio, *CI* confidence interval, *p* *p* value

Correlations are statistically significant for *p* < 0.05 (*) or *p* < 0.01 (**), *p* < 0.001 (***)

Interestingly, cytoplasmic LXR was regarded as an independent prognostic marker in the whole cohort, but this correlation was much stronger in the stage I subgroup, with hazard ratio of 1.871 (95% CI 1.156–3.030; *p* = 0.011) and 7.172 (95% CI 2.764–18.611; *p* = 5.1 × 10^−5^), respectively. In contrast, cytoplasmic LXR did not exhibit an independent prognostic value in the stage II–III subgroup. Similarly, cytoplasmic LXR was a strong independent prognostic marker in the pT1 subgroup (data not shown), but not in the pT2-3 subgroup, nor in the lymph-node negative or positive subgroups.

## Discussion

Based on IHC staining analysis, the present study provides evidence for a differential subcellular distribution of LXR expression in primary BC, and is the first evaluation of its correlations with clinicopathological characteristics and patient survival.

We found that LXR was predominantly expressed in the nuclei of BC cells, but was also detected in their cytoplasm. LXRα expression was previously shown to be predominant in nuclei of hepatocellular carcinoma (Long et al. 2018), as LXRβ isoform in BC tissues (Le Cornet et al. 2020). Consistent with our results, a previous study reported that LXRβ immunoreactivity, not LXRα, was detected both in nuclei and cytoplasm in pancreatic cancer samples, whereas only nuclear staining was present in normal pancreatic ductal epithelial tissues (Candelaria et al. 2014). Besides, LXRβ expression was predominantly localized in cytoplasm of gastric cancer cells (Wang et al. 2019) and of colon cancer cells but in nucleus of normal mucosa cells (Courtaut et al. 2015). In a recent study, only nuclear LXRβ, and not LXRα, was shown to be expressed in the nuclei of 96 triple-negative BC (Pan et al. 2019). In a study focused on LXR intracellular distribution, unliganded LXRα was retained in nucleus whereas unliganded LXRβ was exported to cytoplasm (Prüfer and Boudreaux 2007). In addition, the quick non-genomic activity of LXRβ after activation by ligand induced pyroptosis in cytoplasm of colon cancer cells and then LXRβ translocated into nucleus to initiate transcriptional activity (Derangère et al. 2014). Activation by T0901317, LXRβ translocated into nuclei and inhibited cell proliferation via the Wnt signaling pathway (Wang et al. 2019). Thus, nuclear export of LXR may exist in malignant cells, and intracellular localization of LXR may play different roles in carcinogenesis.

The link between subcellular localization of NR and BC progression seems important parameters in BC etiology. We have previously demonstrated that cytoplasmic PPARγ is predominantly detected in BC tissues and that it is correlated with poor outcome (Shao et al. 2020a). Besides, nuclear THRβ1 in BC tissue appeared to be a marker for poor prognosis, whereas its cytoplasmic form was correlated with favorable survival (Shao et al. 2020b). Transcriptional activity of NRs is mediated by their subcellular localization through a nuclear localization sequence or export sequence. The specific mechanisms and molecular consequences of the cytoplasmic location of various NRs still have to be investigated.

Correlation analysis between LXR expression and clinicopathological parameters indicated that nuclear and total LXR were positively related to ER and PR. A recent study indicated that ER-negative BC had a high transcription response to LXR agonists compared to ER-positive BC (Hutchinson et al. 2019). Besides, 27-hydroxycholesterol acted as not only an LXR ligand but also as an ER agonist. LXR action was accentuated by inhibition of ER signaling (McDonnell et al. 2014). Induction of *Est*, a transcriptional target gene of LXR, decreased estrogen level in mouse model, leading to suppression of BC progression (Gong et al. 2007). LXRα, LXRβ, and their corepressors have been shown to be differentially expressed in ER-positive vs ER-negative BC tumors (Nazih and Bard 2020). Taken together, further study of crosstalk with ER signaling pathway is needed for LXR molecular mechanism in BC biology.

The present study is the first analysis of LXR expression and survival in BC, although correlations between LXR expression and patient outcome have already been analyzed in other cancers. Indeed, in stage II and III non-small-cell lung cancer patients, high LXRα expression was correlated with a favorable outcome, regardless of its subcellular localization (Melloni et al. 2018). Considering the staining of nuclear with/without cytoplasmic forms in human colon cancer, positive LXR was associated with favorable OS (Yun et al. 2017). Moreover, hepatocellular cancer patients with high nuclear LXRα expression had long-term OS (Long et al. 2018). However, BC patients with higher level of LXR ligand target genes expression had an unfavorable outcome compared to those with lower levels (Nguyen-Vu et al. 2013). It is noteworthy that, in our study, cytoplasmic LXR was an independent prognostic factor for poor OS in the whole cohort, although no statistical significance was observed in Kaplan–Meier analysis.

Interestingly, cytoplasmic LXR was strongly correlated with poor OS in the stage I, pT1, and lymph-node negative subgroups. This correlation was confirmed by multivariate analysis in the stage I (and pT1) subgroup, which appeared stronger than that observed in the whole cohort. Therefore, subcellular distribution of LXR expression appears as a parameter that needs to be taken into account in further studies of its mechanism or prognostic value in malignant tumors. Especially for luminal (ER/PR-positive and HER2-negative) early stage BC, the main concern is to identify patients who need to receive also chemotherapy in addition to endocrine therapy (Harbeck et al. 2019). Next to more complex and expensive genomic signatures, IHC assays that enable identification of the subcellular location of relevant therapeutic markers may also be valuable. Cytoplasmic LXR as a strong and independent prognostic marker in the pT1 subgroup may be an excellent candidate to explore.

## Conclusion

In our primary BC cohort, LXR expression, although mostly localized in nuclei, was also detected in the cytoplasm. Cytoplasmic LXR correlated with poor OS in stage I, pT1, and lymph-node negative subgroups. Most importantly, cytoplasmic LXR had a strong and independent prognostic value regarding poor outcome only in early stage primary BC.

## Supplementary Information

Below is the link to the electronic supplementary material.

Supplementary file1 (PPTX 114 KB) Supplemental Figure 1 Kaplan-Meier analyses of patient overall survival (OS) for RXR expression in the whole cohort and different subgroups according to lymph node status, tumor size and Staging. OS curves are presented according to RXR expression, for the whole cohort (A), for stage I (B) and stage II-III (C) subgroups of patients, for lymph node negative (D) or positive (E) subgroups and for the pT1 (F) and pT2-3 (G) subgroups. The optimal IRS cut-off values for RXR expression were determined as 3.5. The number of cases for each group are indicated in each panel.

## Abbreviations

ABCA1: ATP-binding cassette transporter A1
BC: Breast cancer
CI: Confidence interval
DCIS: Ductal carcinoma in situ
ER: Estrogen receptor
FISH: Fluorescence in situ hybridization
HER2: Human epidermal growth factor receptor 2
HR: Hazard ratio
IHC: Immunohistochemistry
IRS: Immunoreactive score
LMU: Ludwig-Maximilians-University
LXR: Liver X receptor
cM: Metastasis
NR: Nuclear receptor
NST: Non-special type
OS: Overall survival
PBS: Non-special type
pN: Lymph-node status
PPAR: Peroxisome proliferator-activated receptor
PR: Progesterone receptor
pT: Tumor size
ROC-curve: Receiver-operating characteristic curve
RXR: Retinoid X receptor
THR: Thyroid hormone receptor

## References

[CR1] Bilotta MT, Petillo S, Santoni A, Cippitelli M (2020) Liver X receptors: regulators of cholesterol metabolism, inflammation, autoimmunity, and cancer. Front Immunol 11:584303. 10.3389/fimmu.2020.584303 33224146 10.3389/fimmu.2020.584303 PMC7670053

[CR2] Bock C et al (2014) Strong correlation between N-cadherin and CD133 in breast cancer: role of both markers in metastatic events. J Cancer Res Clin Oncol 140:1873–1881. 10.1007/s00432-014-1750-z 24962344 10.1007/s00432-014-1750-z PMC11824022

[CR3] Candelaria NR et al (2014) Antiproliferative effects and mechanisms of liver X receptor ligands in pancreatic ductal adenocarcinoma cells. PLoS ONE 9:e106289. 10.1371/journal.pone.0106289 25184494 10.1371/journal.pone.0106289 PMC4153644

[CR4] Chen L, Zhang L, Xian G, Lv Y, Lin Y, Wang Y (2017) 25-Hydroxycholesterol promotes migration and invasion of lung adenocarcinoma cells. Biochem Biophys Res Commun 484:857–863. 10.1016/j.bbrc.2017.02.003 28167281 10.1016/j.bbrc.2017.02.003

[CR5] Chen T, Xu J, Fu W (2020) EGFR/FOXO3A/LXR-α Axis promotes prostate cancer proliferation and metastasis and dual-targeting LXR-α/EGFR shows synthetic lethality. Front Oncol 10:1688. 10.3389/fonc.2020.01688 33224867 10.3389/fonc.2020.01688 PMC7667376

[CR6] Courtaut F et al (2015) Liver X receptor ligand cytotoxicity in colon cancer cells and not in normal colon epithelial cells depends on LXRβ subcellular localization. Oncotarget 6:26651–26662. 10.18632/oncotarget.5791 26450852 10.18632/oncotarget.5791 PMC4694942

[CR7] Derangère V et al (2014) Liver X receptor β activation induces pyroptosis of human and murine colon cancer cells. Cell Death Differ 21:1914–1924. 10.1038/cdd.2014.117 25124554 10.1038/cdd.2014.117 PMC4227150

[CR8] Ditsch N et al (2012) Retinoid X receptor alpha (RXRα) and peroxisome proliferator-activated receptor gamma (PPARγ) expression in breast cancer: an immunohistochemical study. Vivo 26:87–92 22210720

[CR9] Doan TB, Graham JD, Clarke CL (2017) Emerging functional roles of nuclear receptors in breast cancer. J Mol Endocrinol 58:R169-r190. 10.1530/jme-16-0082 28087820 10.1530/JME-16-0082

[CR10] Gong H et al (2007) Estrogen deprivation and inhibition of breast cancer growth in vivo through activation of the orphan nuclear receptor liver X receptor. Mol Endocrinol 21:1781–1790. 10.1210/me.2007-0187 17536009 10.1210/me.2007-0187

[CR11] Harbeck N, Gnant M (2017) Breast cancer. Lancet 389:1134–1150. 10.1016/s0140-6736(16)31891-8 27865536 10.1016/S0140-6736(16)31891-8

[CR12] Harbeck N et al (2019) Breast cancer. Nat Rev Dis Primers 5:66. 10.1038/s41572-019-0111-2 31548545 10.1038/s41572-019-0111-2

[CR13] Heublein S, Mayr D, Meindl A, Kircher A, Jeschke U, Ditsch N (2017) Vitamin D receptor, Retinoid X receptor and peroxisome proliferator-activated receptor γ are overexpressed in BRCA1 mutated breast cancer and predict prognosis. J Exp Clin Cancer Res 36:57. 10.1186/s13046-017-0517-1 28427429 10.1186/s13046-017-0517-1 PMC5399435

[CR14] Hutchinson SA, Lianto P, Roberg-Larsen H, Battaglia S, Hughes TA, Thorne JL (2019) ER-negative breast cancer is highly responsive to cholesterol metabolite signalling. Nutrients. 10.3390/nu11112618 31683867 10.3390/nu11112618 PMC6893441

[CR15] Jalaguier S et al (2017) Complex regulation of LCoR signaling in breast cancer cells. Oncogene 36:4790–4801. 10.1038/onc.2017.97 28414308 10.1038/onc.2017.97 PMC5562849

[CR16] Jeschke U et al (2019) The prognostic impact of the aryl hydrocarbon receptor (AhR) in primary breast cancer depends on the lymph node status. Int J Mol Sci. 10.3390/ijms20051016 30813617 10.3390/ijms20051016 PMC6429124

[CR17] Le Cornet C et al (2020) Circulating 27-hydroxycholesterol and breast cancer tissue expression of CYP27A1, CYP7B1, LXR-β, and ERβ: results from the EPIC-Heidelberg cohort. Breast Cancer Res 22:23. 10.1186/s13058-020-1253-6 32075687 10.1186/s13058-020-1253-6 PMC7031866

[CR18] Lin CY, Gustafsson J (2015) Targeting liver X receptors in cancer therapeutics. Nat Rev Cancer 15:216–224. 10.1038/nrc3912 25786697 10.1038/nrc3912

[CR19] Long H, Guo X, Qiao S, Huang Q (2018) Tumor LXR expression is a prognostic marker for patients with hepatocellular carcinoma. Pathol Oncol Res 24:339–344. 10.1007/s12253-017-0249-8 28508927 10.1007/s12253-017-0249-8

[CR20] Lou R et al (2019) Liver X receptor agonist T0901317 inhibits the migration and invasion of non-small-cell lung cancer cells in vivo and in vitro. Anticancer Drugs 30:495–500. 10.1097/cad.0000000000000758 30724772 10.1097/CAD.0000000000000758 PMC6485493

[CR21] McDonnell DP et al (2014) Obesity, cholesterol metabolism, and breast cancer pathogenesis. Cancer Res 74:4976–4982. 10.1158/0008-5472.Can-14-1756 25060521 10.1158/0008-5472.CAN-14-1756 PMC4167494

[CR22] Melloni G et al (2018) Prognostic role of liver X receptor-alpha in resected stage II and III non-small-cell lung cancer. Clin Respir J 12:241–246. 10.1111/crj.12522 27401614 10.1111/crj.12522

[CR23] Müller K et al (2019) Prognostic relevance of RIP140 and ERβ expression in unifocal versus multifocal breast cancers: a preliminary report. Int J Mol Sci. 10.3390/ijms20020418 30669416 10.3390/ijms20020418 PMC6359229

[CR24] Nazih H, Bard JM (2020) Cholesterol, oxysterols and LXRs in breast cancer pathophysiology. Int J Mol Sci. 10.3390/ijms21041356 32079340 10.3390/ijms21041356 PMC7072989

[CR25] Nelson ER et al (2013) 27-Hydroxycholesterol links hypercholesterolemia and breast cancer pathophysiology. Science 342:1094–1098. 10.1126/science.1241908 24288332 10.1126/science.1241908 PMC3899689

[CR26] Nguyen-Vu T et al (2013) Liver × receptor ligands disrupt breast cancer cell proliferation through an E2F-mediated mechanism. Breast Cancer Res 15:R51. 10.1186/bcr3443 23809258 10.1186/bcr3443 PMC4053202

[CR27] Pan H, Zheng Y, Pan Q, Chen H, Chen F, Wu J, Di D (2019) Expression of LXR-β, ABCA1 and ABCG1 in human triple-negative breast cancer tissues. Oncol Rep 42:1869–1877. 10.3892/or.2019.7279 31432185 10.3892/or.2019.7279 PMC6775801

[CR28] Pommier AJ et al (2010) Liver X Receptor activation downregulates AKT survival signaling in lipid rafts and induces apoptosis of prostate cancer cells. Oncogene 29:2712–2723. 10.1038/onc.2010.30 20190811 10.1038/onc.2010.30

[CR29] Pondé NF, Zardavas D, Piccart M (2019) Progress in adjuvant systemic therapy for breast cancer. Nat Rev Clin Oncol 16:27–44. 10.1038/s41571-018-0089-9 30206303 10.1038/s41571-018-0089-9

[CR30] Prüfer K, Boudreaux J (2007) Nuclear localization of liver X receptor alpha and beta is differentially regulated. J Cell Biochem 100:69–85. 10.1002/jcb.21006 16888799 10.1002/jcb.21006

[CR31] Repa JJ, Mangelsdorf DJ (2000) The role of orphan nuclear receptors in the regulation of cholesterol homeostasis. Annu Rev Cell Dev Biol 16:459–481. 10.1146/annurev.cellbio.16.1.459 11031244 10.1146/annurev.cellbio.16.1.459

[CR32] Scoles DR, Xu X, Wang H, Tran H, Taylor-Harding B, Li A, Karlan BY (2010) Liver X receptor agonist inhibits proliferation of ovarian carcinoma cells stimulated by oxidized low density lipoprotein. Gynecol Oncol 116:109–116. 10.1016/j.ygyno.2009.09.034 19854496 10.1016/j.ygyno.2009.09.034

[CR33] Shao W et al (2020a) Cytoplasmic PPARγ is a marker of poor prognosis in patients with Cox-1 negative primary breast cancers. J Transl Med 18:94. 10.1186/s12967-020-02271-6 32085795 10.1186/s12967-020-02271-6 PMC7035771

[CR34] Shao W et al (2020b) Cytoplasmic and nuclear forms of thyroid hormone receptor β1 are inversely associated with survival in primary breast cancer. Int J Mol Sci. 10.3390/ijms21010330 31947762 10.3390/ijms21010330 PMC6981495

[CR35] Sixou S et al (2018) Importance of RIP140 and LCoR subcellular localization for their association with breast cancer aggressiveness and patient survival. Transl Oncol 11:1090–1096. 10.1016/j.tranon.2018.06.006 30007204 10.1016/j.tranon.2018.06.006 PMC6070698

[CR36] Sung H, Ferlay J, Siegel RL, Laversanne M, Soerjomataram I, Jemal A, Bray F (2021) Global cancer statistics 2020: GLOBOCAN estimates of incidence and mortality worldwide for 36 cancers in 185 countries. CA Cancer J Clin. 10.3322/caac.21660 33538338 10.3322/caac.21660

[CR37] Vedin LL, Lewandowski SA, Parini P, Gustafsson JA, Steffensen KR (2009) The oxysterol receptor LXR inhibits proliferation of human breast cancer cells. Carcinogenesis 30:575–579. 10.1093/carcin/bgp029 19168586 10.1093/carcin/bgp029

[CR38] Wang B, Tontonoz P (2018) Liver X receptors in lipid signalling and membrane homeostasis. Nat Rev Endocrinol 14:452–463. 10.1038/s41574-018-0037-x 29904174 10.1038/s41574-018-0037-x PMC6433546

[CR39] Wang Q et al (2019) Liver X receptor activation reduces gastric cancer cell proliferation by suppressing Wnt signalling via LXRβ relocalization. J Cell Mol Med 23:789–797. 10.1111/jcmm.13974 30338932 10.1111/jcmm.13974 PMC6349166

[CR40] Yun SH, Park MG, Kim YM, Roh MS, Park JI (2017) Expression of chicken ovalbumin upstream promoter-transcription factor II and liver X receptor as prognostic indicators for human colorectal cancer. Oncol Lett 14:4011–4020. 10.3892/ol.2017.6659 28943908 10.3892/ol.2017.6659 PMC5594251

[CR41] Zhang X, Hofmann S, Rack B, Harbeck N, Jeschke U, Sixou S (2017) Fluorescence analysis of vitamin D receptor status of circulating tumor cells (CTCS) in breast cancer: from cell models to metastatic patients. Int J Mol Sci. 10.3390/ijms18061318 28632174 10.3390/ijms18061318 PMC5486139

[CR42] Zhong D, Lyu X, Fu X, Xie P, Liu M, He F, Huang G (2020) Upregulation of miR-124-3p by liver X receptor inhibits the growth of hepatocellular carcinoma cells via suppressing cyclin D1 and CDK6. Technol Cancer Res Treat 19:1533033820967473. 10.1177/1533033820967473 33073697 10.1177/1533033820967473 PMC7592319

