# Supplementary material for: Cytoplasmic LXR expression is an independent marker of poor prognosis for patients with early stage primary breast cancer
Source: J Cancer Res Clin Oncol. 2021 Jun 3;147(9):2535–44. doi: 10.1007/s00432-021-03670-y (PMC8310839; doi:10.1007/s00432-021-03670-y)
Supplement: Supplementary file 1 — Supplementary file1 (PPTX 114 KB) Supplemental Figure 1 Kaplan-Meier analyses of patient overall survival (OS) for RXR expression in the whole cohort and different subgroups according to lymph node status, tumor size and Staging. OS curves are presented according to RXR expression, for the whole cohort (A), for stage I (B) and stage II-III (C) subgroups of patients, for lymph node negative (D) or positive (E) subgroups and for the pT1 (F) and pT2-3 (G) subgroups. The optimal IRS cut-off values for RXR expression were determined as 3.5. The number of cases for each group are indicated in each panel. [file 432_2021_3670_MOESM1_ESM.pptx]

## Slide 1
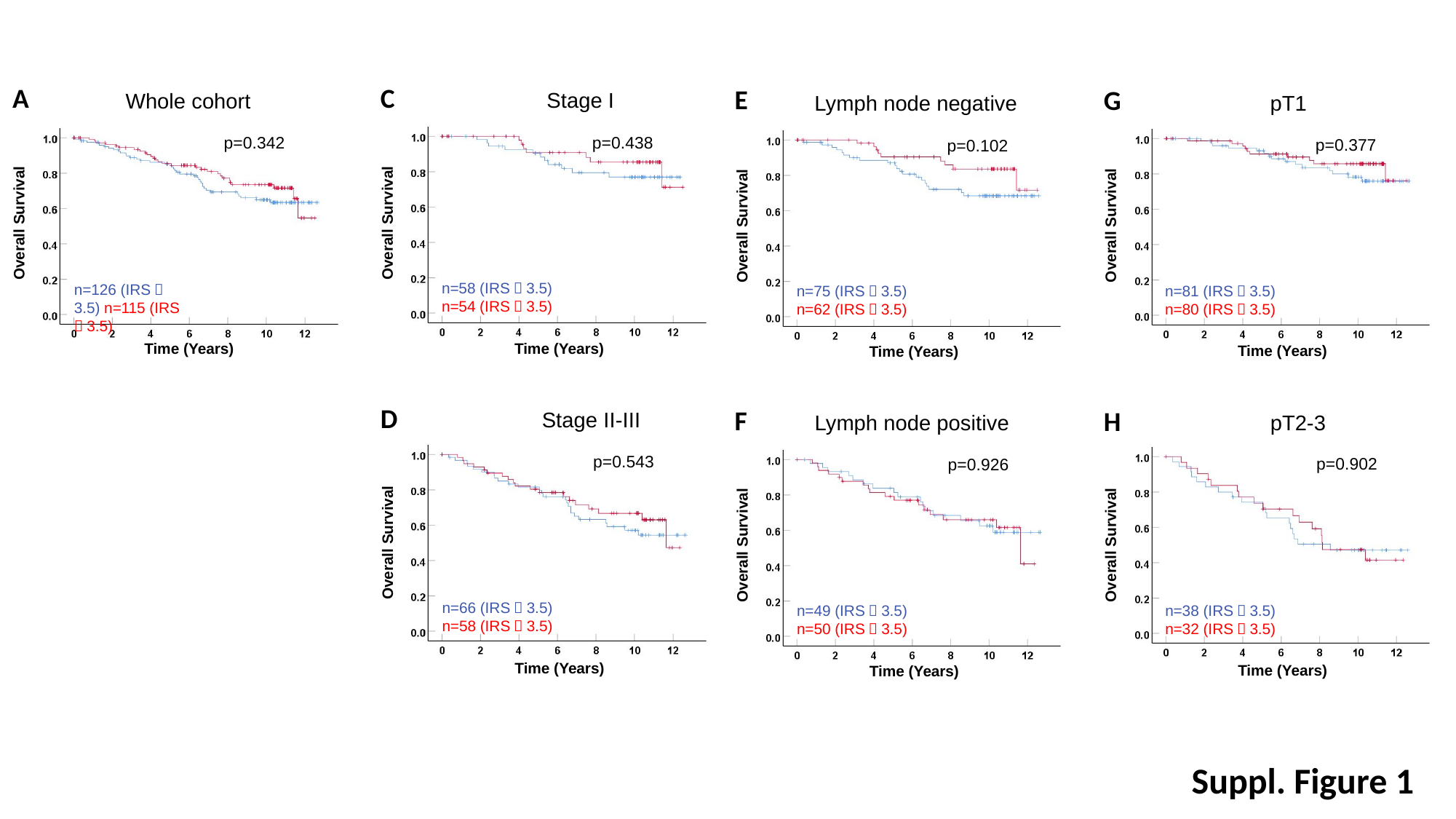

A
C
E
G
Stage I
Whole cohort
pT1
Lymph node negative
p=0.342
p=0.438
p=0.377
p=0.102
Overall Survival
Overall Survival
Overall Survival
Overall Survival
n=58 (IRS＜3.5) n=54 (IRS＞3.5)
n=126 (IRS＜3.5) n=115 (IRS＞3.5)
n=81 (IRS＜3.5) n=80 (IRS＞3.5)
n=75 (IRS＜3.5) n=62 (IRS＞3.5)
Time (Years)
Time (Years)
Time (Years)
Time (Years)
D
F
H
Stage II-III
pT2-3
Lymph node positive
p=0.543
p=0.902
p=0.926
Overall Survival
Overall Survival
Overall Survival
n=66 (IRS＜3.5) n=58 (IRS＞3.5)
n=38 (IRS＜3.5) n=32 (IRS＞3.5)
n=49 (IRS＜3.5) n=50 (IRS＞3.5)
Time (Years)
Time (Years)
Time (Years)
Suppl. Figure 1
